# Usual Interstitial Pneumonia Preceding Collagen Vascular Disease: A Retrospective Case Control Study of Patients Initially Diagnosed with Idiopathic Pulmonary Fibrosis

**DOI:** 10.1371/journal.pone.0094775

**Published:** 2014-04-15

**Authors:** Masato Kono, Yutaro Nakamura, Noriyuki Enomoto, Dai Hashimoto, Tomoyuki Fujisawa, Naoki Inui, Masato Maekawa, Takafumi Suda, Thomas V. Colby, Kingo Chida

**Affiliations:** 1 Second Division, Department of Internal Medicine, Hamamatsu University School of Medicine, Hamamatsu, Japan; 2 Department of Laboratory Medicine, Hamamatsu University School of Medicine, Hamamatsu, Japan; 3 Department of Laboratory Medicine and Pathology, Mayo Clinic, Scottsdale, Arizona, United Sates of America; Medical University of South Carolina, United States of America

## Abstract

**Background:**

The aim of this study was to evaluate the cumulative incidence and the predictive factors for collagen vascular disease (CVD) in patients initially diagnosed with idiopathic pulmonary fibrosis (IPF), and to examine the features of patients who then developed CVD.

**Methods:**

This was a retrospective review of 111 consecutive patients with IPF diagnosed at our institution. None of the patients fulfilled any of the CVD criteria from the American College of Rheumatology (ACR) within 6 months or more after the diagnosis of IPF.

**Results:**

Ten patients (9.0%) developed CVD during the follow-up period: four had rheumatoid arthritis (RA); four had microscopic polyangiitis (MPA); one had systemic sclerosis (SSc); and one had SSc and Sjogren’s syndrome (SjS). The mean time until CVD diagnosis was 3.9 years. The cumulative incidences of CVD at 1, 5, and 10 years were 0.91%, 9.85%, and 15.5%, respectively. Patients who developed CVD were significantly younger, more likely to be women and had a better prognosis than those with IPF. Cox proportional hazards regression analysis showed that female sex and the presence of lymphoid aggregates with germinal centers were significantly associated with the occurrence of CVD in patients initially diagnosed with IPF.

**Conclusions:**

CVD is an important underlying condition in IPF, and shows better prognosis. The possibility of the development of CVD should remain a consideration in the follow-up of IPF.

## Introduction

Interstitial pneumonia (IP) commonly complicates collagen vascular disease (CVD) [1]–[5], and it is well known that IP may be the first or only manifestation of CVD [6]. None of these patients with IP fulfill the diagnostic criteria for defined CVDs, and most may be diagnosed as idiopathic interstitial pneumonias (IIPs) [7], [8]. It has been reported that patients with IIPs cannot be distinguished from those with IP associated with CVD (CVD-IP) before the systemic signs of CVD appear [6]. Although we sometimes encounter patients who fulfill the criteria for a CVD in the clinical course of IIP, the cumulative incidence and predictive factors associated with the occurrence of CVD remain unclear.

In patients with IIPs, non-specific interstitial pneumonia (NSIP) has been reported to be associated with autoimmune diseases including CVDs [9]–[11], and there are some reports of idiopathic NSIP preceding the diagnosis of CVD [11]–[14]. However, there are a few reports of patients who fulfill the criteria for a CVD after the diagnosis of idiopathic pulmonary fibrosis (IPF)/usual interstitial pneumonia (UIP) [4], [15]. Recently, some patients with CVD-associated signs and symptoms who nonetheless failed to fulfill the criteria defined CVDs have been diagnosed using new criteria, such as those for undifferentiated connective tissue disease (UCTD) [16], lung-dominant connective tissue disease (LD-CTD) [17], and autoimmune-featured interstitial lung disease (AIF-ILD) [18]. However, these criteria are not always fulfilled in patients with IIPs preceding the diagnosis of CVD.

Here, we evaluated the cumulative incidence of CVD and the clinical features of patients who fulfilled the criteria for any CVD after an initial diagnosis of IPF. Furthermore, we examined the predictive factors associated with the development of CVD in patients initially diagnosed with IPF.

## Patients and Methods

### Patients and Diagnostic Criteria

We studied 155 consecutive patients with IPF who were diagnosed clinically or underwent surgical lung biopsy (SLB) at our institution from 1990 through 2007. The diagnosis of IPF was based on a history, physical examination, high-resolution computed tomography (HRCT) findings and/or histologic examination, and the appropriateness of the IPF diagnosis in each case was retrospectively reevaluated according to the current international diagnostic criteria [19]. Within at least 6 months of the initial diagnosis, none of the patients fulfilled the American College of Rheumatology (ACR) criteria defining CVDs, including rheumatoid arthritis (RA) [20], polymyositis/dermatomyositis (PM/DM) [21], systemic sclerosis (SSc) [22], systemic lupus erythematosus (SLE) [23] and Sjogren’s syndrome (SjS) [24], or the Chapel Hill Consensus Conference criteria defining systemic vasculitis, including microscopic polyangiitis (MPA) [25]. The study protocol was approved by the Ethical Committee of Hamamatsu University School of Medicine. Patient approval and/or informed consent were waived because the study involved a retrospective review of patient records, images and pathologies. Our institutional review board determined that ethical approval was not necessary and did not require the patient’s approval or informed consent.

### Data Collection

Clinical data were obtained from patients’ medical records. Laboratory findings, pulmonary function test results and bronchoalveolar lavage (BAL) data obtained at the time of the initial diagnosis were also recorded. For the patients who developed CVD, additional clinical data were obtained at the time of the CVD diagnosis. The duration of time until the CVD diagnosis and the cumulative incidence of CVD among patients with IPF were calculated.

### High-resolution Computed Tomography (HRCT)

HRCT examinations of the lungs were performed on 1.0- or 1.5-mm-thick sections to evaluate radiographic abnormalities at the time of initial diagnosis. The HRCT images were based on previously published international criteria for IPF [19]. We reviewed the presence and the distribution of each of the following signs: consolidation, ground-glass opacity (GGO), reticulation, centrilobular nodules, honeycombing, traction bronchiectasis, lymph node enlargement, and cysts.

### Pathological Review

Histological classification was based on the previously published criteria for IIPs and IPF [7], [19]. A diagnosis of UIP was originally made histologically by a lung pathologist at our facility. Lung biopsy specimens were also reviewed by a second lung pathologist (T. V. C.) who was unaware of the clinical or physical findings. The degree of each of the following pathologic findings was scored semiquantitatively (absent, 0; mild, 1; moderate, 2; marked, 3): alveolar inflammation, fibrosis, intra-alveolar macrophages, organizing pneumonia, fibroblastic foci, and lymphoid aggregates with germinal centers. For lymphoid aggregates, the case was scored as 1 when there was one germinal center and as 3 when there were six or more in a low-power field (2×). The presence or absence of each of the following pathologic findings was also assessed: honeycombing, extensive pleuritis, prominent plasmacytic infiltration, and dense perivascular collagen including the histopathological features of the proposed provisional criteria for LD-CTD [17].

### Statistical Methods

For two-group comparisons involving binary data, we used the chi-square test. Comparisons involving continuous data were made using the Mann-Whitney *U*-test. The cumulative survival probabilities and the cumulative incidence of CVD were estimated using the Kaplan-Meier method. The log-rank test was used to compare survival among the groups of patients. Cox proportional hazards regression analysis was used to identify significant variables predicting the development of CVD. Statistical analyses were performed using JMP Start Statistics (SAS Institute Inc., NC, USA). A *P* value <0.05 was considered significant.

## Results

### Patient Characteristics

**Figure 1:**
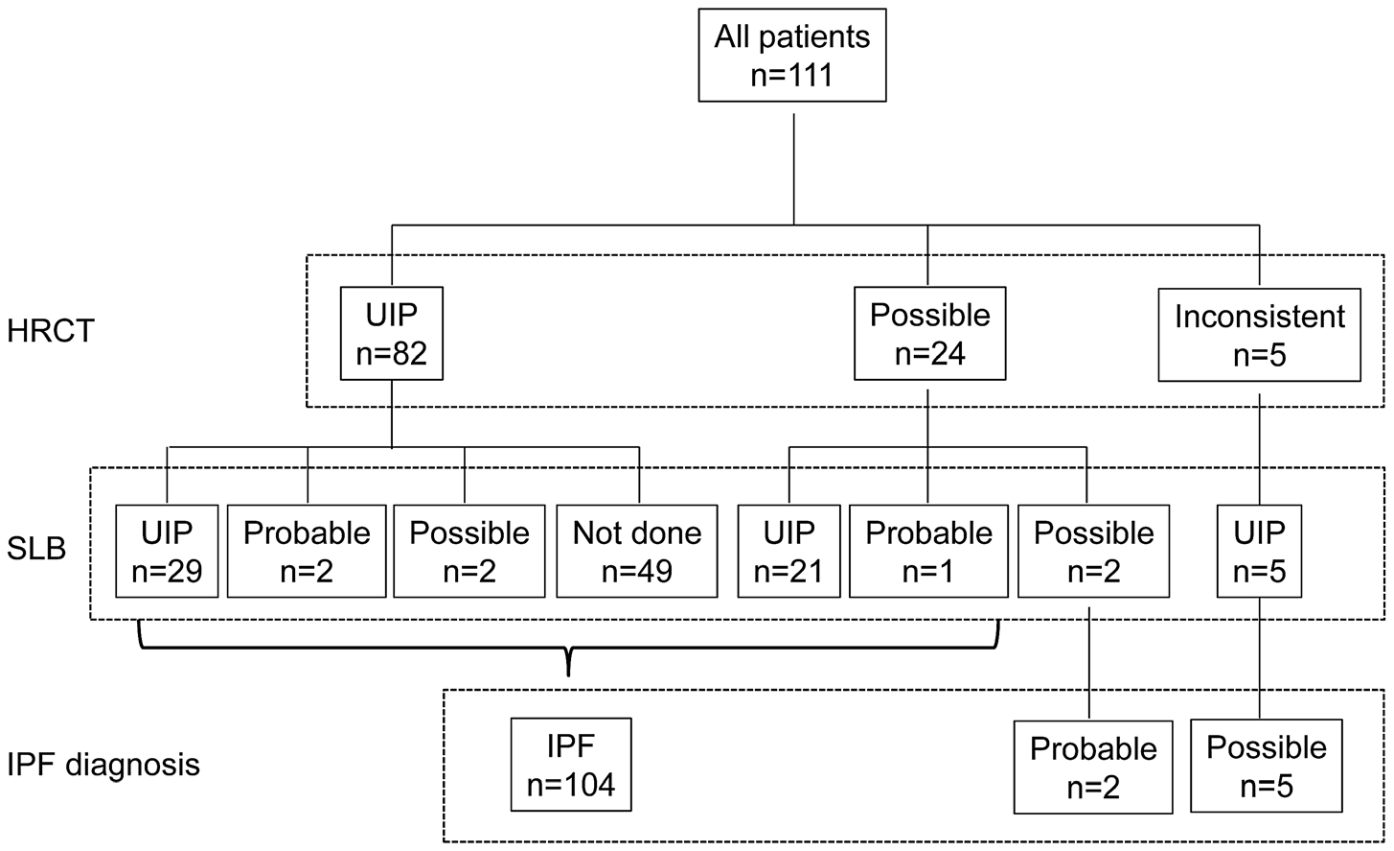
The algorithm for IPF diagnosis among enrolled patients. All 111 patients were diagnosed with IPF (definite, n = 104; probable, n = 2; possible, n = 5) according to the algorithm for IPF diagnosis. HRCT, high-resolution computed tomography; SLB, surgical lung biopsy; IPF, idiopathic pulmonary fibrosis; UIP, usual interstitial pneumonia.

Of the original 155 patients, 44 patients were excluded because of inadequate clinical information. The remaining 111 patients were included in this study. All of them were diagnosed with IPF (definite, n = 104; probable, n = 2; possible, n = 5) according to HRCT and/or the histopathological pattern [19] (**Figure 1**), and the clinical characteristics of these patients are shown in **Table 1**. During the observation periods (6.4±4.9 years), 10 patients (9%) developed CVD (all patients were initially diagnosed with definite IPF). We compared the clinical characteristics of patients who developed CVD and those with IPF at the initial diagnosis (**Table 1**). Compared to patients with IPF, those who developed CVD were younger and more often female. There were no statistically significant differences in smoking habits, observation period, or the rate of SLB between groups. Between-group comparisons revealed no significant difference in serum levels of lactate dehydrogenase (LDH), creatine phosphokinase (CPK), C-reactive protein (CRP), KL-6, surfactant protein-D (SP-D), PaO_2_ in room air, or pulmonary function. BAL was performed in 83 patients (74.8%): in nine of 10 who developed CVD and in 74 of 101 who did not. The patients who developed CVD exhibited a reduced percentage of lymphocytes (*P* = 0.04) in comparison to those with IPF, but no other BAL findings were significantly different between the groups.

**Table 1:** Patient characteristics.

Characteristics	All patients	CVD-IP	IPF	*P* value
**No. of cases**	111	10	101	
**Age, yr**	64.7±9.4	59.6±8.0	65.2±9.3	0.03
**Gender, male/female**	99/12	5/5	94/7	<0.001
**Smoking habit, n Current/former/never**	41/56/14	2/5/3	39/51/11	n. s.
**Observation period, yr**	6.4±4.4	8.7±5.9	6.1±4.2	n. s.
**Surgical lung biopsy, n (%)**	63 (56.8)	7 (70.0)	56 (55.4)	n. s.
**Initial IPF diagnosis, n definite/probable/possible**	104/2/5	10/0/0	94/2/5	n. s.
**Laboratory data**				
** LDH, IU/L**	253±85	280±89	251±84	n. s.
** CPK, IU/L**	90±48	101±62	89±46	n. s.
** CRP, mg/dL**	0.94±2.97	0.42±0.40	0.98±3.08	n. s.
** KL-6, U/mL**	1129±739	1707±1408	1096±694	n. s.
** SP-D, ng/mL**	273±195	357±179	269±196	n. s.
** PaO_2_ on room air, Torr**	79.5±14.9	79.3±11.0	79.4±15.2	n. s.
**Pulmonary function tests**				
** FVC, % predicted**	84.2±20.9	83.7±21.7	84.2±20.9	n. s.
** FEV_1.0_, % predicted**	81.9±9.2	84.0±4.3	81.7±9.6	n. s.
** DLco, % predicted**	84.5±25.1	86.4±30.6	84.2±24.7	n. s.
**Bronchoalveolar lavage (BAL)** *				
** Macrophages, %**	90.6±11.3	94.5±3.7	90.1±11.8	n. s.
** Lymphocytes, %**	6.2±8.0	3.0±3.5	6.6±8.3	0.04
** Neutrophils, %**	1.6±6.0	1.4±1.5	1.7±6.3	n. s.
** Eosinophils, %**	1.4±4.7	0.6±0.9	1.5±5.0	n. s.
** CD4/CD8 ratio**	2.8±2.4	3.4±2.6	2.7±2.5	n. s.

CVD, connective vascular disease; IPF, idiopathic pulmonary fibrosis; CVD-IP, interstitial pneumonia associated with collagen vascular disease; LDH, lactate dehydrogenase; CPK, creatine phosphokinase; CRP, C-reactive protein; SP-D, surfactant protein-D; FVC, forced vital capacity; FEV_1.0_, forced vital capacity in 1 second; DLco; diffusion capacity for carbon monoxide; n. s., not significant.

*BAL was performed in 83 patients (74.8%): CVD-IP (n = 9), IPF (n = 74).

The clinical characteristics of the patients who developed CVD at the time of CVD diagnosis are shown in **Table 2**. These 10 patients included four patients with RA (40%), four patients with MPA (40%), one patient with SSc (10%), and one patient with overlap syndrome (SSc/SjS) (10%). This group included five women (50%). Age at the time of CVD diagnosis was 63.2±7.4 years. The average time from the initial diagnosis of IPF to the CVD diagnosis was 3.9±2.4 years. None of the patients had undergone treatment for IP with corticosteroids and/or immunosuppressive agents (ISA) prior to the development of CVD. Each had been diagnosed with CVD based on their symptoms and other findings associated with CVD. All four patients who developed RA showed polyarthralgia and positivity for rheumatoid factor (RF). In the four patients who developed MPA, two patients (Cases 7, 8) had alveolar hemorrhage, three patients (Cases 6, 7, 8) showed renal dysfunction with hematuria, two patients (Cases 5, 8) exhibited a purpuric rash with small-vessel vasculitis detected by skin biopsy, and all four patients showed positivity for myeloperoxidase antineutrophil cytoplasmic antibody (MPO-ANCA). Both patients who developed SSc showed Raynaud’s phenomenon and scleroderma, and one patient (Case 10) also showed dry mouth with inflammation of the salivary glands associated with SjS. One patient died secondary to the acute exacerbation (AE) of IP (Case 6) and one patient died of unknown cause (Case 10). The deaths occurred at 5.8 and 3.8 years, respectively, from the initial diagnosis.

**Table 2:** Characteristics of the patients who had developed CVD at the time of CVD diagnosis.

Case	Age, yr	Sex, F/M	CVD	SLB	Duration until CVD diagnosis	Findings associated with CVD diagnosis	Treatment before CVD diagnosis	Outcome
1	63	F	RA	UIP	4yr4mo	Arthralgia	None	Alive
2	60	F	RA	UIP	2yr10mo	Arthralgia, Muscular pain	None	Alive
3	62	M	RA	UIP	1yr1mo	Arthralgia	None	Alive
4	66	F	RA	UIP*	4yr4mo	Arthralgia	None	Alive
5	64	M	MPA	UIP	3yr7mo	Rash (vasculitis)	Macrolide	Alive
6	76	M	MPA	None	6mo	Renal dysfunction	None	Dead
7	65	M	MPA	None	8yr	Alveolar hemorrhage, Renal dysfunction	None	Alive
8	66	F	MPA	None	4yr5mo	Alveolar hemorrhage, Rash (vasculitis), Renal dysfunction	None	Alive
9	64	M	SSc	UIP	7yr5mo	Raynaud, scleroderma	None	Alive
10	46	F	SSc/SjS	UIP	3yr	Raynaud, scleroderma, Dry mouth	None	Dead

CVD, connective vascular disease; yr, year; F, female; M, male; SLB, surgical lung biopsy; RA, rheumatoid arthritis; MPA, microscopic polyangiitis; SSc, systematic sclerosis; SjS, Sjogren’s syndrome; UIP, usual interstitial pneumonia; mo, month.

*SLB was performed at the time of RA diagnosis.

### Systemic Findings Associated with CVD and Autoantibodies

We compared the systemic findings associated with CVD and autoantibodies at the time of the initial diagnosis in patients who developed CVD and in those with a persistent diagnosis of IPF (**Table 3**). Anti-nuclear antibody (ANA) and RF test results were considered positive with a result ≥1+ or a titer of 1∶40 and ≥15 IU/mL, respectively. No systemic findings associated with CVD or autoantibodies recorded at the time of the initial diagnosis differed between groups. About half of the patients with IPF had positive ANA results owing to the low cutoff level for ANA. In the patients who developed CVD, there were no positive findings of CVD-specific autoantibodies at the time of the initial diagnosis, except for ANA and RF. However, all patients who developed CVD had systemic findings associated with CVD and/or autoantibodies at the time of the CVD diagnosis. In particular, ANA was positive in 8/10 patients (80.0%) and RF was positive in 9/10 patients (90.0%) at the time of the CVD diagnosis.

**Table 3:** Systemic findings and autoantibodies in the patients who developed CVD and in those with IPF at the time of initial diagnosis.

Characteristics	CVD-IP	IPF	*P* value
**Systemic findings**			
** Raynaud’s phenomenon**	1 (10.0)	3 (2.9)	n. s.
** Arthralgias/joint swelling**	0	3 (2.9)	n. s.
** Photosensitivity**	0	0	n. s.
** Unintentional weight loss**	1 (10.0)	2 (1.9)	n. s.
** Morning stiffness**	0	3 (2.9)	n. s.
** Dry eye or dry mouth**	0	3 (2.9)	n. s.
** Dysphagia**	0	0	n. s.
** Recurrent unexpected fever**	0	0	n. s.
** Gastroesophageal reflex**	0	1 (1.0)	n. s.
** Skin change (rash)**	0	3 (2.9)	n. s.
** Oral ulceration**	0	0	n. s.
** Non-androgenic alopecia**	0	0	n. s.
**Autoantibodies, n (%)**			
** Anti-nuclear antibody**	4/10 (40.0)	46/91 (50.5)	n. s.
** Rheumatoid factor**	3/9 (33.3)	19/85 (22.3)	n. s.
** Anti-SCL 70 antibody**	0/5 (0)	0/55 (0)	n. s.
** Anti-SSA antibody**	0/5 (0)	1/57 (1.7)	n. s.
** Anti-SSB antibody**	0/5 (0)	0/56 (0)	n. s.
** Anti-Jo1 antibody**	0/4 (0)	0/45 (0)	n. s.
** Anti-centromere antibody**	0/4 (0)	1/36 (2.7)	n. s.
** Anti-RNP antibody**	0/5 (0)	0/40 (0)	n. s.
** Anti-DNA antibody**	0/8 (0)	1/74 (1.3)	n. s.
** MPO-ANCA**	0/3 (0)	4/54 (7.4)	n. s.
** PR3-ANCA**	0/3 (0)	1/49 (1.9)	n. s.

CVD, connective vascular disease; IPF, idiopathic pulmonary fibrosis; CVD-IP, interstitial pneumonia associated with collagen vascular disease; MPO-ANCA, myeloperoxidase antineutrophil cytoplasmic antibody; PR3-ANCA, proteinase 3-antineutrophil cytoplasmic antibody; n. s., not significant.

### HRCT and Pathological Findings

The HRCT and pathological findings in the patients who developed CVD and in those with IPF are shown in **Table 4**. The HRCT findings showed that there was no statistically significant difference between the groups with regard to the presence or distribution of each finding, including consolidation, GGO, reticulation, centrilobular nodules, honeycombing, traction bronchiectasis, lymph node enlargement, and cysts, at the initial diagnosis.

**Table 4:** HRCT and pathological findings in the patients who developed CVD and in those with IPF at the time of initial diagnosis.

Characteristics	CVD-IP	IPF	*P* value
**HRCT findings**			
** Subpleural, basal predominance, %**	100	97.3	n.s.
** Consolidation, %**	20.0	11.7	n.s.
** GGO, %**	40.0	28.7	n.s.
** Reticulation, %**	100	98.9	n.s.
** Centrilobular nodules, %**	20.0	6.4	n.s.
** Honeycombing, %**	90.0	72.3	n.s.
** Traction bronchiectasis, %**	80.0	66.0	n.s.
** Lymph node enlargement, %**	10.0	5.3	n.s.
** Cysts, %**	40.0	26.6	n.s.
**Pathological findings**			
** Alveolar inflammation** *	1.41±0.58	1.30±0.51	n.s.
** Fibrosis** *	2.00±1.10	2.11±0.80	n.s.
** Intra-alveolar macrophages** *	0.83±0.75	1.21±0.73	n.s.
** Organizing pneumonia** *	0.33±0.81	0.13±0.33	n.s.
** Fibroblastic foci** *	0.75±0.52	1.08±0.60	n.s.
** Honeycombing, %**	83.3	62.5	n.s.
** Lymphoid aggregates with germinal centers** *	1.17±1.17	0.18±0.47	<0.01
** Extensive pleuritis, %**	16.7	10.7	n.s.
** Prominent plasmacytic infiltration, %**	16.7	12.5	n.s.
** Dense perivascular collagen, %**	0	1.8	n.s.

HRCT, high-resolution computed tomography; CVD, connective vascular disease; IPF, idiopathic pulmonary fibrosis; CVD-IP, interstitial pneumonia associated with collagen vascular disease; GGO, ground-glass opacity; n. s., not significant.

*scoring of the severity: absent 0, mild 1, moderate 2 and marked 3.

**Figure 2:**
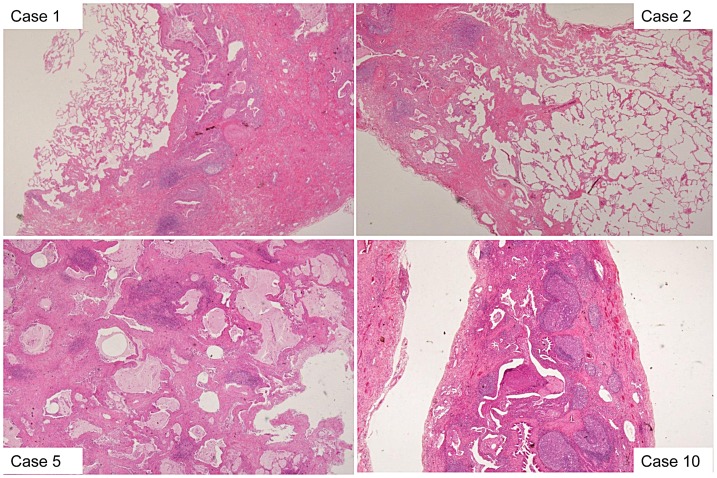
Pathological findings in the patients who developed CVD (Cases 1, 2, 5, 10). All patients showed a pathological UIP pattern at the time of the initial diagnosis. Lymphoid aggregates with germinal centers were prominent in the patients who developed CVD.

SLB was performed in 63 patients (56.8%): in seven of 10 who developed CVD and in 56 of 101 who did not. One patient who developed CVD (Case 4) was excluded from the statistical analysis because she had undergone SLB at the time of the RA diagnosis; this patient had fulfilled the clinical/radiologic criteria for IPF at the time of the initial diagnosis. The other six patients who developed CVD underwent SLB before the CVD diagnosis. Regarding each pathological finding listed in **Table 4**, the patients who developed CVD had significantly more lymphoid aggregates with germinal centers (*P*<0.01) than did patients with IPF. However, there were no statistically significant differences in the scores for alveolar inflammation, fibrosis, intra-alveolar macrophages, organizing pneumonia, fibroblastic foci, the presence of honeycombing, extensive pleuritis, prominent plasmacytic infiltration, or dense perivascular collagen between the groups. The pathological findings in the patients who developed CVD (Cases 1, 2, 5, 10) are shown in **Figure 2**. Interestingly, the pathological findings for one patient who underwent SLB at the time of the RA diagnosis (Case 4) showed a UIP pattern with prominent lymphoid follicles.

### Treatment and Outcome

All of the patients who developed CVD were treated with corticosteroids alone or in combination with ISA therapy after the diagnosis of CVD. They were treated significantly more often than the patients with IPF (*P* = 0.01) and also had a significantly higher rate of treatment with corticosteroids alone (*P* = 0.01) (**Table 5**). Two of the patients who developed MPA (Cases 5, 7) were treated with corticosteroids and cyclophosphamide. Patients remaining in the IPF group had a higher rate of treatment with other agents, such as pirfenidone and N-acetylcysteine (NAC), than those who developed CVD. The patients who developed CVD had a higher rate of AE, although the difference was not statistically significant, and had significantly higher rates of overall death and death due to a respiratory cause (*P* = 0.04, *P* = 0.02, respectively).

**Table 5:** Treatment and outcome throughout the course of the patients who developed CVD and those with IPF.

	CVD-IP	IPF	*P* value
**Treatment for IP, n (%)**	10 (100)	61 (60.4)	0.01
** Corticosteroids alone, n (%)**	8 (80.0)	12 (11.8)	<0.01
** Corticosteroids+ISA, n (%)**	2 (20.0)	34 (33.6)	n. s.
** Others, n (%)**	0 (0)	23* (22.7)	0.09
**Acute exacerbation, n (%)**	1 (10.0)	30 (29.7)	n. s.
**Death due to respiratory failure, n (%)**	1 (10.0)	43 (42.5)	0.02
**Overall death, n (%)**	2 (20.0)	53 (52.4)	0.04

CVD, collagen vascular disease; IPF, idiopathic pulmonary fibrosis; CVD-IP, interstitial pneumonia associated with collagen vascular disease; ISA, immune suppressive agents; n. s., not significant.

*Pirfenidone (n = 14), N-acetylcysteine (NAC) (n = 5), intravenous immunoglobulin (IVIG) (n = 2), Pirfenidone+NAC (n = 1), Pirfenidone+IVIG (n = 1).

**Figure 3:**
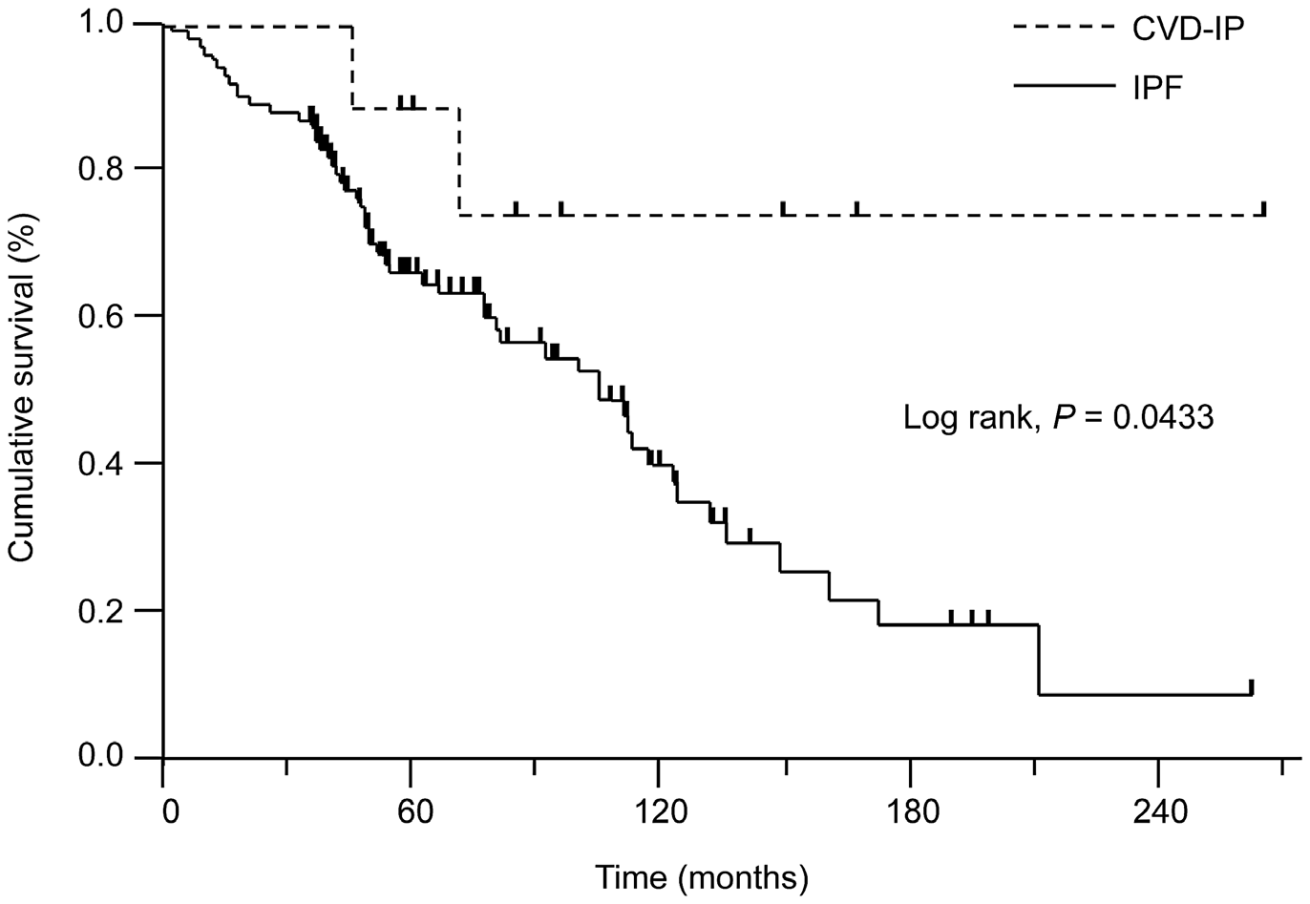
Survival curves for the patients who developed CVD and those with IPF. Patients who developed CVD had a significantly better survival rate than those with IPF (log-rank, *P* = 0.0433). IPF, idiopathic pulmonary fibrosis; CVD-IP, interstitial pneumonia associated with collagen vascular diseaseG.

A comparison of the survival curves from the time of initial diagnosis for patients who developed CVD and those who did not is shown in **Figure 3**. The former had a significantly better survival rate than the latter (log-rank test: *P*<0.05).

### Cumulative Incidence of CVD and Predictive Factors for the Development of CVD in Patients with IPF

**Figure 4:**
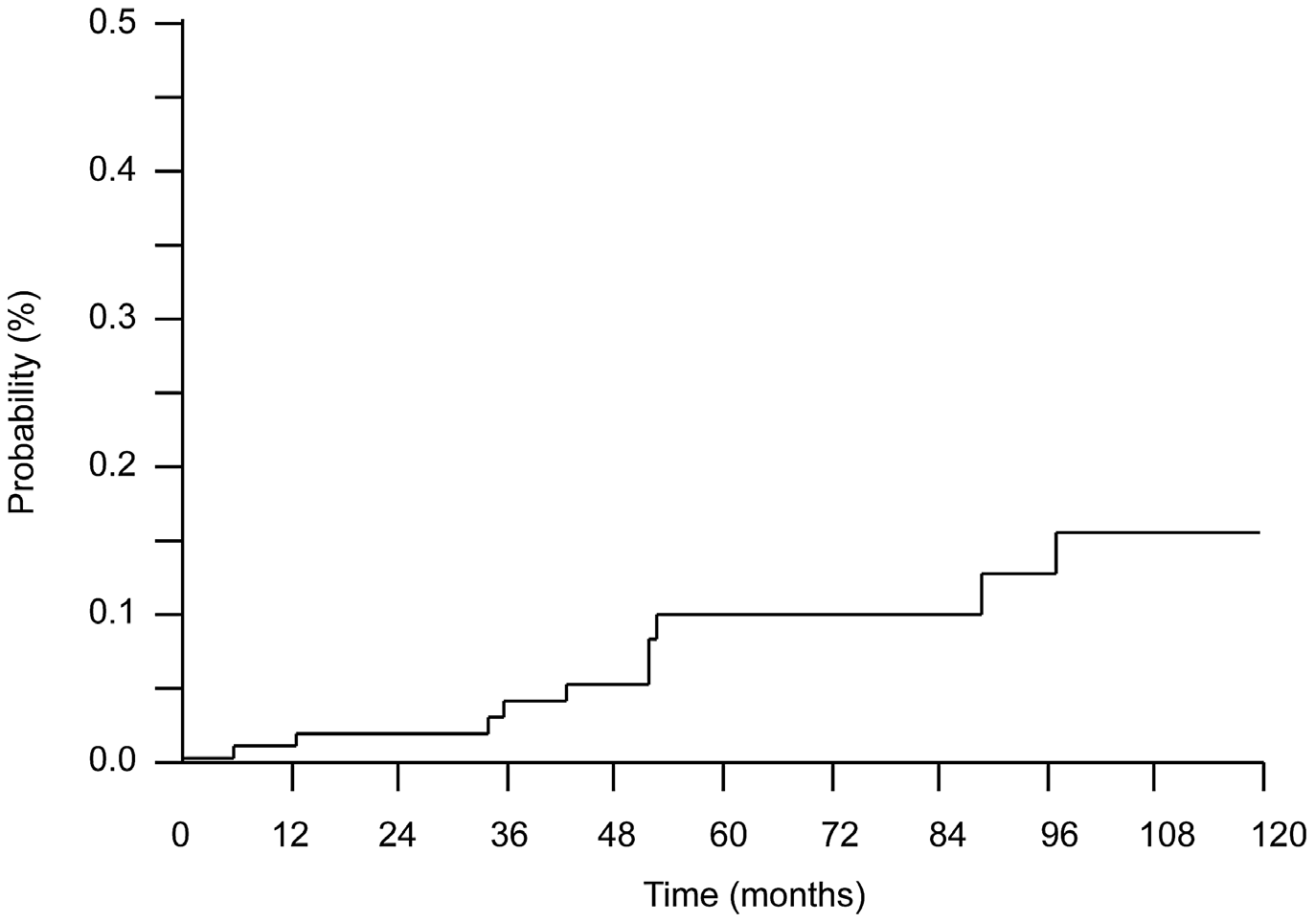
Cumulative incidence of CVD among patients with IPF. Cumulative incidences of CVD at 1, 5, and 10 years were 0.91%, 9.85%, and 15.5%, respectively.

A Kaplan-Meier plot of the cumulative incidence of CVD is shown in **Figure 4**. The cumulative incidences of CVD at 1, 5, and 10 years were 0.91%, 9.85%, and 15.5%, respectively.

Factors predictive for the development of CVD at the initial diagnosis are shown in **Table 6**. Cox proportional hazards regression analysis showed that being female [hazard ratio 3.333, 95% confidence interval (CI) 0.732–6.441, *P*<0.01] and the score for lymphoid aggregates with germinal centers (hazard ratio 3.367, 95% CI 1.503–7.434, *P*<0.01) were significantly associated with the development of CVD in patients with IPF.

**Table 6:** Predictive factors for the development of CVD in patients with IPF, according to univariate Cox proportional hazards analysis.

Variables	Hazard ratio	95% CI		*P* value
		Lower	Upper	
**Age, year**	0.955	0.891	1.023	0.189
**Sex, female**	3.333	1.732	6.441	<0.01
**Symptoms associated with CVD** * **, positive**	1.325	0.514	2.656	0.502
**Autoantibodies** ** **, positive**	1.216	0.597	3.152	0.614
**KL-6, U/mL**	1.001	0.999	1.002	0.297
**SP-D, ng/mL**	1.001	0.996	1.004	0.566
**FVC, % predicted**	0.987	0.959	1.018	0.412
**BAL lymphocytes, %**	0.891	0.649	1.028	0.168
**HRCT findings**				
** Consolidation, positive**	1.196	0.278	2.801	0.747
** GGO, positive**	1.239	0.568	2.416	0.556
** Centrilobular nodules, positive**	1.360	0.315	3.196	0.593
** Lymph node enlargement, positive**	1.341	0.311	3.136	0.607
**Pathological findings**				
** Alveolar wall inflammation, score**	1.572	0.296	7.199	0.576
** Fibroblastic foci, score**	0.458	0.092	1.931	0.296
** Honeycombing, positive**	0.574	0.131	1.433	0.258
** Lymphoid aggregates with germinal centers, score**	3.367	1.503	7.434	<0.01

CVD, collagen vascular disease; IPF, idiopathic pulmonary fibrosis; CI, confidence interval; SP-D, surfactant protein-D; FVC, forced vital capacity; BAL, bronchoalveolar lavage; HRCT, high-resolution computed tomography; GGO, ground-glass opacity.

*one or more positive systemic symptoms in Table 2.

**one or more positive autoantibodies in Table 3.

## Discussion

In this retrospective study, we evaluated the cumulative incidence and the predictive factors for CVD in patients with IPF, and examined the features of patients who subsequently fulfilled the criteria for any CVD. The rate of development of CVD was 9% in consecutive patients initially diagnosed with IPF during the observation period, and the cumulative incidences of CVD at 1, 5, and 10 years were 0.91%, 9.85%, and 15.5%, respectively. It was difficult to predict the latency of CVD in patients with IPF based on systemic findings and the presence of autoantibodies, but Cox proportional hazards regression analysis showed that female sex and prominent lymphoid aggregates with germinal centers were significantly associated with the development of CVD in patients with IPF.

Pulmonary manifestation including IP commonly complicates CVD and increases the mortality and morbidity associated with CVD [26], [27]. IP may be the first or only recognized manifestation of a CVD, and it is estimated to affect approximately 15–20% of patients with IIPs [6], [9], [10], [26]–[28]. The development of CVD after an IIP diagnosis was documented best for SSc and PM/DM patients. It has been reported that IP precedes PM/DM in about 40% of patients [29]. It was also reported that SSc sine scleroderma (ssSSc)-associated IP might be present in IIP patients [30], developing in some cases into typical SSc [31].

In patients with IIPs, NSIP is reportedly associated with autoimmune disease, including CVD [9]–[11]. Previous studies have documented the development of CVD in patients with NSIP [11]–[14]. Sato *et al*. reported a small series of seven patients with histologically proven NSIP who later developed CVDs (PM/DM n = 4, SSc n = 1, RA n = 1, MPA n = 1) more than 6 months after the first presentation of NSIP [12]. Recently, in a large retrospective analysis, 3–10% of patients with idiopathic NSIP developed CVDs [13], [14], with variable intervals between CVD occurrences (9 months to 8 years) [13].

However, there have been only a few reports of patients who developed CVD in presumed cases of IPF/UIP [4], [15]. Lee *et al*. examined the histopathologic patterns and clinical features of 18 patients with RA-IP, and reported three patients in whom IP preceded the diagnosis of RA (1.6 years, 2.5 years, 7 years): two patients had UIP and one had NSIP [4]. Van der Kamp *et al*. reported three patients with IP as the first manifestation of SSc, and 1 of these three patients had biopsy-proven UIP 4 years prior to the SSc diagnosis [15]. The present study revealed that RA and MPA were the main CVDs that developed after the initial IP diagnosis. In the patients with IP-associated RA or MPA, the UIP pattern is seen more commonly than other pathological patterns [4], [5], [32]. CVD development after an IIP diagnosis might vary according to the histological pattern of the patient’s IIP.

Although it was reported that younger patients with IPF, especially women, without clinical or serologic features at presentation may subsequently manifest clinical features of CVD [19], IIP patients could not be distinguished from CVD-IP patients before systemic signs of CVD appeared [6], [12], [13]. Our data showed similar results, but the pathological findings of lymphoid aggregates with germinal centers had clinical significance as a predictive factor of occult CVD in patients with IPF. Song *et al*. compared the pathological difference between CVD-UIP (most patients had RA and SSc) and IPF/UIP, and showed by multivariate analysis that germinal center score was the most discriminating factor for CVD-UIP [33]. Cipriani *et al*. also evaluated the difference between CVD-UIP and IPF/UIP by quantitative pathological assessment, and found that there was a trend toward more and larger lymphoid aggregates in the RA-UIP subset [34]. Our finding suggests that an UIP pattern with prominent germinal centers may be associated with subsequent CVD in IPF patients.

Some patients with IIPs have symptoms and/or autoantibodies suggestive of autoimmune disorders without fulfilling the criteria for defined CVDs [8]. Such patients can now be diagnosed based on new criteria, including those for UCTD [16], LD-CTD [17] and AIF-ILD [18]. We could speculate that patients exhibiting certain characteristics of CVD would subsequently fulfill the disease’s official diagnostic criteria. However, in this study, there was no statistically significant difference between the patients who developed CVD and the remaining patients with IPF in the frequency with which patients fulfilled these criteria (UCTD, LD-CTD and AIF-ILD) at the time of the initial diagnosis (data not shown), suggesting that patients without symptoms of signs suggestive of CVD can develop CVD.

It has been reported that CVD-IP has a better prognosis than IIP [35], [36]. The difference in outcome appears to be related to a better prognosis for CVD-UIP than IPF/UIP, rather than to the higher prevalence of NSIP [35], [36]. In addition, the improved prognosis of CVD-IP could be attributable to the fact that subclinical or non-progressive IP is common. In contrast with IPF patients, those with CVD are more likely to be administered anti-inflammatory or immune-suppressive treatment, which might affect survival [27]. Our results also revealed that IPF patients who developed CVD (i.e., CVD-IP) had significantly better survival than those who did not, although some reports showed that there was no prognostic difference between IPF/UIP and IPF accompanied by the occult or probable form of CVD (i.e., antibody-positive IPF or LD-CTD) [33], [37]. In terms of survival, IPF patients in our series survived longer (median survival time was 8.4 years; 5-year survival rate was 66.4%) than those in most other series [19]. The difference might be due to the fact that the patients included in our series had relatively early-stage disease. Further study including prospective research is needed in this regard.

Several recent reports showed that RA-UIP had a worse prognosis than the other histological patterns of RA-IP [36], [38]–[40]. However, it is uncertain whether RA-UIP and IPF have equally poor prognoses. Indeed, in this study, the patients who developed CVD including RA-UIP had a better prognosis than did other patients with IPF. All the patients with RA-UIP were treated with corticosteroids and exhibited a slowly progressive course of disease without acute exacerbation during the observation period.

The present study has several limitations. First, this was a retrospective study from a single institution, and some patients were excluded because of inadequate information. In addition, the IPF patients in our series were at a relatively early stage of disease and had longer survival than those in most other series. These were selection and recall biases. Second, although the present study included a relatively large number of patients with IPF, the sample size was too small to determine the precise cumulative incidence and clinical characteristics of those who developed CVD. Third, not all patients underwent SLB or were examined for all of the CVD-specific antibodies. Most patients could not be tested for new autoantibodies such as anti-cyclic citrullinated peptide (CCP) antibody and anti-aminoacyl-tRNA synthetase (ARS) antibody, and so on. In addition, the assays for identifying certain autoantibodies had changed during the study period. Fourth, it might be possible for the development of CVD to be suppressed by the use of immunomodulation drugs or an early observation period, including early death.

In conclusion, we suggest that some CVDs are an underlying condition in IPF and may have a better prognosis than IPF. Although it was unclear whether IP was actually the first manifestation of CVD or an incidentally coexisting disorder, it is very important to perform careful follow-up to watch for the development of CVD, especially RA, MPA, and SSc-associated diseases, in patients diagnosed with IPF.
